# Rhizosphere bacteria community and functions under typical natural halophyte communities in North China salinized areas

**DOI:** 10.1371/journal.pone.0259515

**Published:** 2021-11-11

**Authors:** Fating Yin, Fenghua Zhang, Haoran Wang

**Affiliations:** 1 Agricultural College, Shihezi University, Shihezi City, China; 2 Key Laboratory of Oasis Eco-agriculture, Xinjiang Production and Construction Corps, Shihezi University, Shihezi, Xinjiang, China; Shandong University, CHINA

## Abstract

Soil salinity is a serious environmental issue in arid China. Halophytes show extreme salt tolerance and are grow in saline-alkaline environments. There rhizosphere have complex bacterial communities, which mediate a variety of interactions between plants and soil. High-throughput sequencing was used to investigated rhizosphere bacterial community changes under the typical halophyte species in arid China. Three typical halophytes were *Leymus chinensis* (LC), *Puccinellia tenuiflora* (PT), *Suaeda glauca* (SG). The dominant phyla were *Proteobacteria*, *Actinobacteria*, *Chloroflexi*, *Gemmatimonadetes*, *Acidobacteria* and *Bacteroidetes*, *Suaeda glauca* rhizosphere has stronger enrichment of *Nitrospirae* and *Cyanobacteria*. The Ace, Chao and Shannon indices were significantly higher in soils under LC and SG (*P*<0.05). Functional predictions, based on 16S rRNA gene by PICRUSt, indicated that Energy metabolism, Amino acid metabolism, Carbohydrate metabolism and Fatty acid metabolism are dominant bacterial functions in three halophytes rhizosphere soil. Carbon metabolism, Oxidative phosphorylation, Methane metabolism, Sulfur metabolism and Nitrogen metabolism in SG were significantly higher than that in LC and PT. Regression analysis revealed that rhizosphere soil bacterial community structure is influenced by soil organic matter (SOM) and soil water content (SWC), while soil bacterial community diversity is affected by soil pH. This study contributes to our understanding of the distribution characteristics and metabolic functions under different halophyte rhizosphere bacterial communities, and will provide references for the use of rhizosphere bacteria to regulate the growth of halophytes and ecological restoration of saline soil.

## Introduction

Soil salinity is one of the rising environmental issues causing considerable yield losses worldwide especially in arid and semiarid regions [1]. It damages the soil structure, reduces soil quality and limits the growing of crops [2]. Halophytes, such as *Suaeda glauca* (Bunge) Bunge, *Puccinellia tenuiflora*, *Tamarix chinensis Lour* and *Leymus chinensis* are plant species that grown well in saline soil due to their saline-alkali tolerance features [3, 4]. They contribute enormously to the supply of food, fuel, fiber and fodder in developing countries [5]. Saline soils are thought harsh environments for life, but such environments survive active and diverse microbes [6]. Soil microbes are crucial to the maintenance of ecosystem functions due to their contributions to soil structure formation and stability, organic matter decomposition, and nutrition cycling [7]. Bacterial communities were determined by both salt content and pH in saline soils under halophytic vegetation, and the effect of salt content was similar to that of the soil pH [8]. Although most studies on halophytes have focused on phytoremediation of saline land and heavy metal contaminated soils, little known distribution characteristics and metabolic functions of soil bacteria in the rhizosphere under different halophytes [9, 10].

The rhizosphere is a critical interface supporting the exchange of nutrients between plants and their associated soil environment [11]. Bacteria are the most abundant and diverse group of microbes in the soil, and major drivers of biogeochemical cycles and participate in maintaining ecosystem functioning [12]. Many studies indicated that halotolerant rhizobacteria isolated from halophytes enhance salt tolerance of their host plants [13]. For example, Marasco et al. (2016) reported that rhizobacteria colonized in the *Salicornia strobilacea* rhizoplane is capable of improving plant growth [14]. Kearl et al. (2019) found that several bacteria communities, such as *Halomonas*, *Bacillus*, and *Kushneria*, can improve the growth of alfalfa under saline conditions [15]. Likewise, several studies have been published on beneficial effects of bacterial application on wheat growth under salt conditions [16]. However, more information on the bacterial community present from the rhizospheric of various halophytes is still needed before these halotolerant rhizobacteria can be applied in salinity affected agriculture soil [17].

The high-throughput sequencing technology has promoted our understanding of the bacterial community composition, diversity and in relation to their environments [18]. PICRUSt has been widely used to predict the soil bacterial functions based on the16S rRNA gene [19]. Here, we investigated the composition of rhizosphere soil bacterial communities under various halophyte species using high-throughput sequencing in the north China. The objectives of this study were to: (i) reveal the bacterial community composition, diversity and predicted functions in rhizospheric soil under different halophyte species, (ii) to determine the possible factors in shaping bacterial community changes in these rhizospheric soils.

## Material and methods

### Site description, experimental design and sampling

Rhizosphere samples were collected from three typical salinized areas in the north of China, no permits were required (40°28’37"-44°34’19" N, 85°54’03"-123°17’45" E). The three typical salinized areas are located in Tumochuan Plain in Inner Mongolia Autonomous Region, Songnen Plain in Jilin Province, and Manasi River Basin in the Xinjiang Province. The dominant halophyte is *Leymus chinensis* (Trin.) Tzvel in Tumochuan Plain, *Puccinellia tenuiflora* (Griseb.) Scribn. et Merr. in Songnen Plain, and *Suaeda glauca* (Bunge) Bunge in Manasi River Basin (more than 85% of species present). *Leymus chinensis* has good nutritional value and high palatability, which is a kind of forage for local livestock [20]. *Puccinellia tenuiflora* is monocotyledonous halophyte and an alkali tolerant species that can survive in highly alkaline soil [21]. *Suaeda glauca* is a rigid, annual, 100 cm high grass occurring in alkali conditions such as coastal region, wastelands, canal banks, and fields and tender plants are delicious and edible [22]. A description of each sites is given in Table 1.

**Table 1:** General description of geography at the three experimental sites.

Sites	LC	PT	SG
Dominat halophyte	*Leymus chinensis* (Trin.) Tzvel.	*Puccinellia tenuiflora* (Griseb.) Scribn. et Merr	*Suaeda glauca* (Bunge) Bunge
Coordinates	40°28’37", 111°04’32"	45°59’12", 123°02’13"	44°37’53", 85°55’36"
Mean sea level (m)	1000	130	450
Climate	Temperate semi-arid continental monsoon climate	Temperate semi-arid continental monsoon climate	Temperate continental climate
Mean annual precipitation (mm)	400	399	<300
Mean annual evaporation (mm)	1000–1100	662	>1000
Mean annual temperature (°C)	6.3	5.2	6.5
Soil classification	Sandy soil	Meadow soil	Sandy soil

LC, *Leymus chinensis* (Trin.) Tzvel.; PT, *Puccinellia tenuiflora* (Griseb.) Scribn. et Merr.; SG, *Suaeda glauca* (Bunge) Bunge.

Rhizosphere soil samples were collected in the middle of May 2019. Within an area of approximately 35 ha, we randomly selected nine plots (replicates) in each sample, each plot 3 m×3 m within monospecific population of each halophyte species for sampling. For rhizosphere sampling, the roots were gently shaken to remove the loosely adhering soil. The soil attached to the roots, which is considered to be the rhizosphere soil, was collected by using sterile brushes [23]. Rhizosphere samples of each replicate were put in individual sterile plastic bags and transported to the laboratory immediately. After visible stones and plant residues were removed, rhizosphere soil was sieved (2 mm mesh) and separated into two subsamples, one portion was air-dried for the determination of chemical analysis, and the reminder was stored in a -20°C refrigerator for molecular analysis.

### Soil properties determination

Soil pH was measured with a compound electrode (INESA Scientific PHSJ-3F) using a soil to water ratio of 1:5 [24]. Electrical conductivity (EC) was measured using a soil-water suspension (1: 2.5 soil-water ratio) [25]. Soil water content (SWC) was measured after drying in an oven at 105°C for 24 h [26]. Soil organic matter (SOM) was determined by oxidizing organic C with potassium dichromate (K_2_Cr_2_O7) [27].

### DNA extraction and PCR amplification

Soil DNA was extracted from 0.5 g fresh subsamples using Soil DNA Kit (Omega Bio-Tek Inc., Norcross, GA, USA), according to the manufacturer’s protocols. The quality of extracted DNA was assessed by 1% agarose gel electrophoresis and substandard samples were extracted again until all the samples passed the quality control. All extracted DNA samples were stored at -20°C for further analysis. The V4-V5 hypervariable regions of the soil bacterial 16S rRNA gene were subjected to high-throughput sequencing by Majorbio Pharmaceutical Technology Co., Ltd. (Shanghai, China) using PE300 sequencing platform (Illumina, Inc., San Diego, CA, USA). The V4-V5 bacterial 16S rRNA gene were amplified by PCR using the primers pair 515 F/907R. The PCR program was as follows: denaturation at 95°C for 30 s; annealing at 5°C for 30 s; extension at 72°C for 45 s; 27 cycles; holding at 72°C for 10 min and storing at 10°C.

In order to guarantee the accuracy of the analysis results, Quantitative Insights into Microbial Ecology (QIIME software; version1.9.0) was used for sequence filtering, and then the chimeric sequence was removed using Mothur software to obtain a high-quality sequence for subsequent analysis [28]. The qualified sequences were clustered into operational taxonomic units (OTUs) with a 97% similarity cut-off using the Usearch program (version 7.0; http://drive5.com/uparse/). The taxonomy of each OTU was analyzed by RDP classifier algorithm against the Silva (SSU123) 16S rRNA database (SILVA ribosomal RNA database project; Max Planck Institute for Marine Microbiology and Jacobs University, Bremen, Germany) using confidence threshold of 70%.

### Data processing and statistical analysis

Venn diagrams were used to compare the similarities between soil bacterial communities at OTU level. According to the species abundance of each sample in the OTU list, soil bacterial richness indices (Chao and Ace) and diversity index (Shannon) were calculated by using Mothur software [29]. Analysis of similarity (ANOSIM) and nonmetric multidimensional scaling (NMDS) based on the unweighted UniFrac distance were used to reveal changes in soil bacterial community structures [30]. The influences of soil properties on soil bacterial community structures were examined by Mantel test and distance-based redundancy analysis (Db-RDA). The influences of soils properties on soil bacterial community structures were examined by Linear Regression. PICRUSt (Phylogenetic Investigation of Communities by Reconstruction of Unobserved States) was performed to predict the abundance of main metabolic function genes [19]. The functional genes were predicted from the Kyoto Encyclopedia of Genes and Genomes (KEGG) catalogue [26].

SPSS version 20.0 (SPSS Inc., Chicago, USA) was utilized to data statistics and analysis of variance to determine the difference between soil properties, bacterial community and bacterial function spectrum. R was used to perform and drawn Venn diagram, NMDS, ANOSIM, db-RDA and Linear Regression analysis [31]. The specific functional differences were performed using Origin version 9.0 (Microcal Software, Inc., Northampton, MA, USA).

## Results

### Soil properties

The changes of rhizosphere soil properties under different halophytes were shown in Table 2. Significant differences of soil pH were found between rhizosphere samples from the different halophytes (*P* < 0.05, Tukey’s test). PT (*Puccinellia tenuiflora* (Griseb.) Scribn. et Merr.) had the highest soil pH, whereas LC (*Leymus chinensis* (Trin.) Tzvel.) had the lowest pH value. Soil EC significantly (*P* < 0.05) varied from 0.45 to 1.44 (mS·cm^-1^) across the rhizosphere samples, and LC hadthe highest values. There was a significant variation in SOM under different halophytes rhizosphere (*P* < 0.05). The highest SOM content was found in rhizosphere samples from LC, which was 84.31% higher than that of SG (*Suaeda glauca* (Bunge) Bunge.). SWC followed the order PT > LC > SG, and there was no significant difference between LC and PT (*P* > 0.05).

**Table 2:** Soil physicochemical properties under three halophytes.

Rhizosphere samples	pH	EC (mS·cm^-1^)	SOM (g·kg^-1^)	SWC
LC	9.22±0.10	1.44±0.42	36.13±3.80	0.22±0.02
PT	10.53±0.03	0.45±0.04	20.45±7.94	0.22±0.05
SG	9.54±0.43	0.47±0.08	5.67±2.37	0.06±0.05

LC, *Leymus chinensis* (Trin.) Tzvel.; PT, *Puccinellia tenuiflora* (Griseb.) Scribn. et Merr.; SG, *Suaeda glauca* (Bunge) Bunge.

EC—electrical conductivity; SOM—soil organic matter; SWC—soil water content. Values are means ± standard deviation. Different lowercase letters into each column are significantly different (Tukey’s test, *P* < 0.05).

### Soil bacterial community composition and diversity

**Fig 1:**
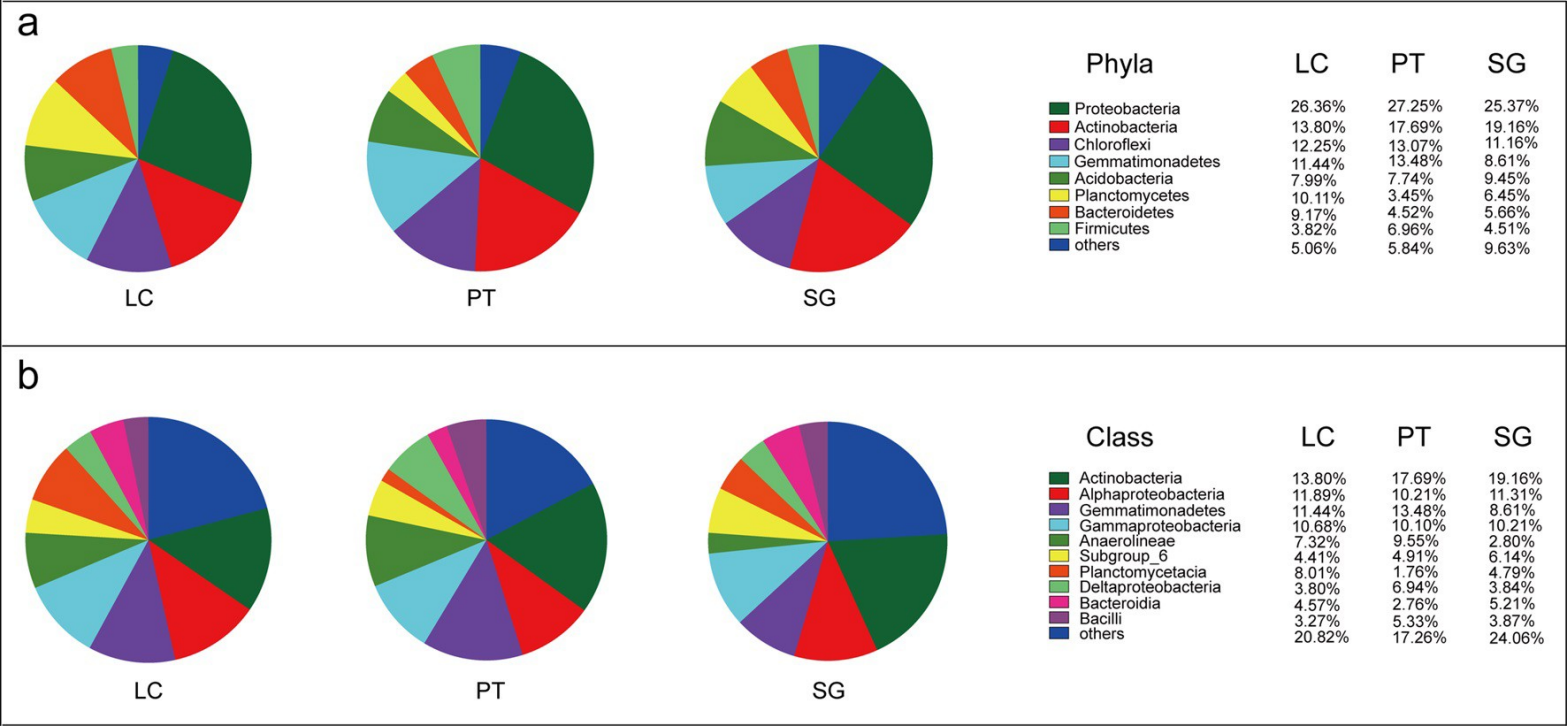
Relative abundance of rhizosphere soil bacterial communities at the phylum level (a) and class level (b) across three halophytes. LC, *Leymus chinensis* (Trin.) Tzvel.; PT, *Puccinellia tenuiflora* (Griseb.) Scribn. et Merr.; SG, *Suaeda glauca* (Bunge) Bunge.

A total of 38 bacterial phyla were observed from rhizosphere samples. *Proteobacteria*, *Actinobacteria*, *Chloroflexi*, *Gemmatimonadetes*, *Acidobacteria*, *Bacteroidetes*, *Planctomycetes*, and *Firmicutes* were dominant phyla through entire soil samples (Fig 1A). The highest relative abundance of *Proteobacteria*, *Chloroflexi and Gemmatimonadetes* were observed in PT, which were 27.25%, 13.07% and 13.48%, respectively. However, the lowest relative abundance of *Proteobacteria* (25.37%), *Chloroflexiand* (11.16%) *and Gemmatimonadetes* (8.61%) were found in SG, and the relative abundance of *Actinobacteria* (19.16%) and *Acidobacteria* (9.45%) were highest in three rhizosphere samples. The relative abundance of *Planctomycetes*, *Entotheonellaeota* and *Hydrogenedentes* in LC were significantly higher than that in PT and SG (S1a Fig, *P* < 0.05, Tukey’s test). The relative abundance of *Nitrospirae*, *Cyanobacteria*, *Deinococcus-Thermus*, *Armatimonadetes* and *BRC1* in SG were significantly higher than those of LC and PT. To further compare the differences in bacterial community composition between rhizosphere samples under different halophytes, we conducted the relative abundance of bacteria (> 0.02%) at the class level (Fig 1B). The dominant classes were *Actinobacteria*, *Alphaproteobacteria*, *Gemmatimonadetes*, *Gammaproteobacteria* and *Anaerolineae* in LC, PT and SG. The relative abundance of *Planctomycetacia* and *Rhodothermia* in LC were significantly higher than that in PT and SG (S1b Fig, *P* < 0.05). The relative abundance of *Anaerolineae*, *Deltaproteobacteria* and *NC10* in PT were significantly higher than that in LC and SG. The relative abundance of *Chloroflexia*, *Nitrospira*, *Oxyphotobacteria*, *Deinococci* and *Gitt-GS-136* in SG, which were significantly higher than of LC and PT.

Venn diagrams indicated that the sum of total observed OTUs in the rhizosphere samples from three halophytes was 10,154 (S2 Fig), and 1,413 OTUs were shared by all three groups. The numbers of OTUs co-occurred in LC, PT and SG were 1,232, 522 and 931, respectively.

**Fig 2:**
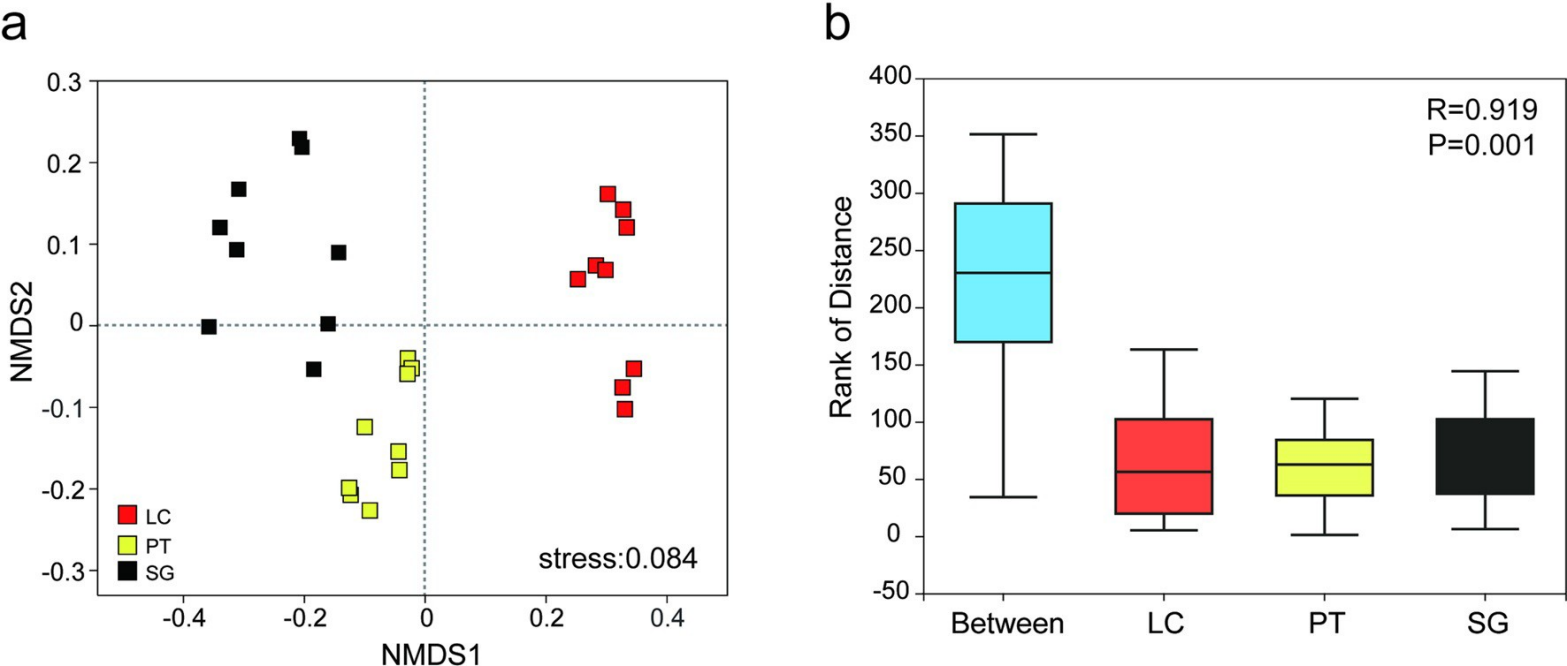
NMDS (a) and ANOSIM (b) analysis based on the unweighted UniFrac distances at OTU level. LC, *Leymus chinensis* (Trin.) Tzvel.; PT, *Puccinellia tenuiflora* (Griseb.) Scribn. et Merr.; SG, *Suaeda glauca* (Bunge) Bunge.

To determine whether bacterial community structure was associated with alteration of different halophytes rhizosphere samples, we profiled the overall structural changes of bacterial community by using NMDS based on unweighted UniFrac dissimilarities (Fig 2A). NMDS ordinations showed rhizosphere samples from three regions are accumulated together respectively, samples in PT and SG separated with LC along NMDS 1. The ANOSIM results showed that the differences in the bacterial community structure of three halophytes rhizosphere samples are greater than the differences within the groups (Fig 2B, R = 0.82, *P* = 0.001).

**Fig 3:**
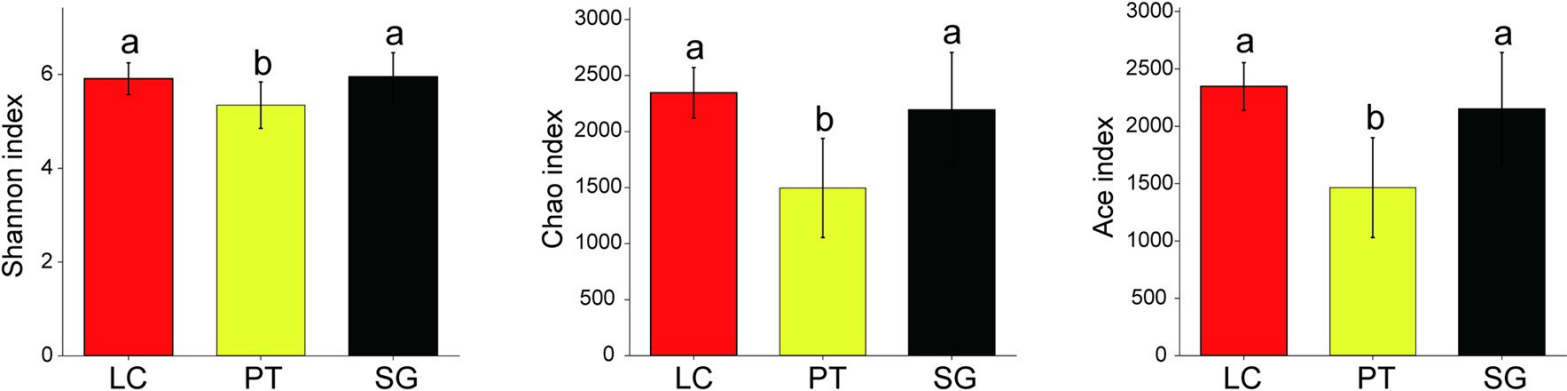
Bar chart for alpha diversity of the rhizosphere soil bacterial communities under three halophytes. Different lowercase letters into each column are significantly different (Tukey’s test, *P* < 0.05). LC, *Leymus chinensis* (Trin.) Tzvel.; PT, *Puccinellia tenuiflora* (Griseb.) Scribn. et Merr.; SG, *Suaeda glauca* (Bunge) Bunge.

Soil bacterial diversity is essential for measuring soil micro-ecological health. Shannon, Ace and Chao indices in PT were significantly lower than that in LC and SG (Fig 3, *P* < 0.05), but there was no significant difference between PT and SG (*P* > 0.05).

### Correlation between bacterial communities and soil properties

**Fig 4:**
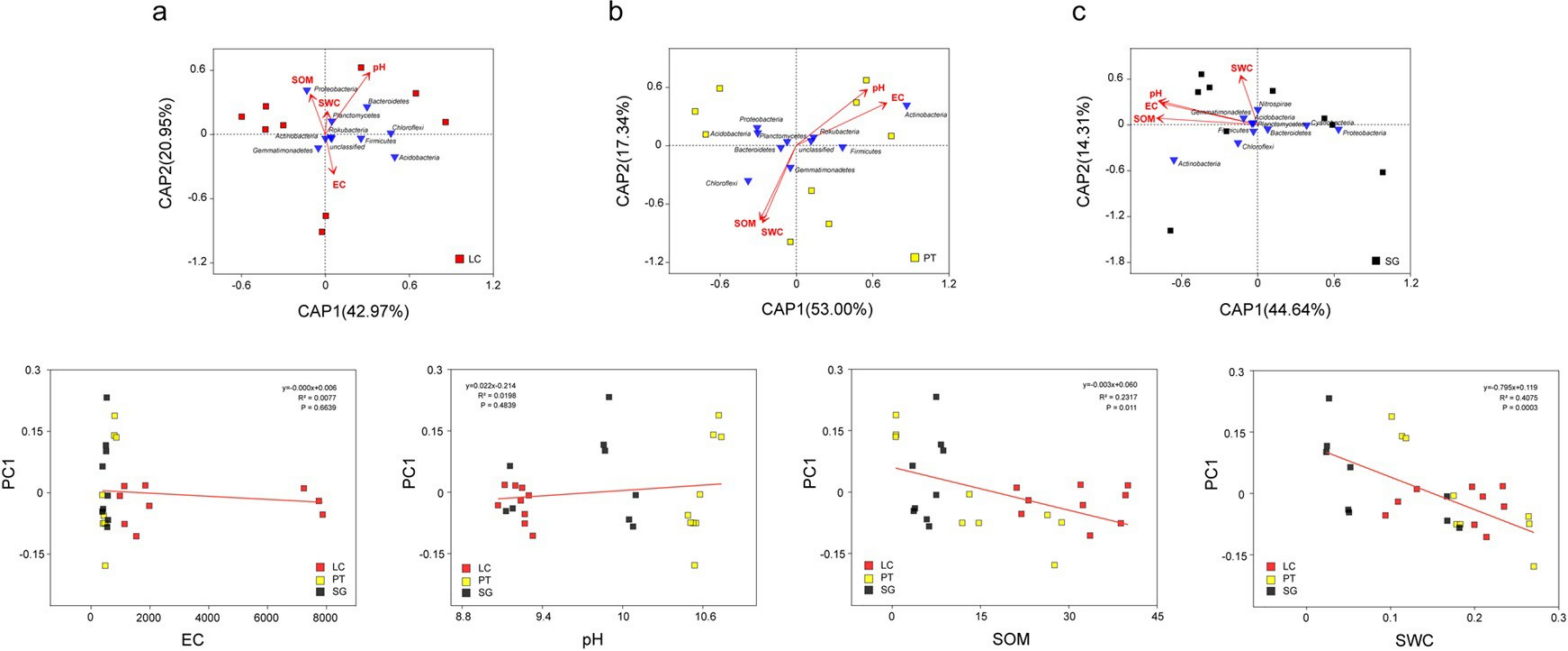
RDA analysis between bacterial communities (phylum level) and soil properties of LC (a), PT (b) and SG (c). Linear regression analysis at the phylum level between the PCA1 and pH, EC, SOM, SWC. SOM—soil organic matter; SWC—soil water content. LC, *Leymus chinensis* (Trin.) Tzvel.; PT, *Puccinellia tenuiflora* (Griseb.) Scribn. et Merr.; SG, *Suaeda glauca* (Bunge) Bunge.

**Fig 5:**
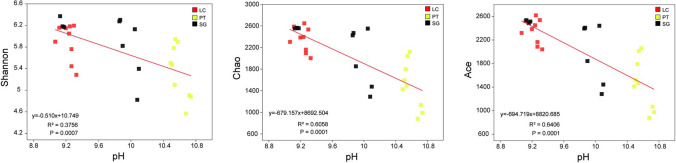
Linear regression analysis between the alpha diversity indices and pH. LC, *Leymus chinensis* (Trin.) Tzvel.; PT, *Puccinellia tenuiflora* (Griseb.) Scribn. et Merr.; SG, *Suaeda glauca* (Bunge) Bunge.

Db-RDA analysis was used to study the influence of soil properties on bacterial community composition at phylum level (Mantel test, *R* = 0.28, *P* = 0.01). It was difference that the effects of three rhizosphere soil properties on rhizosphere bacterial communities (Fig 4). It is apparent from LC’s chart that the two axes of CAP axis explain 63.92% of total variation (Fig 4A), and soil pH, EC, SOM and SWC in LC were No correlation with the CAP axis (S1 Table). Rhizosphere soil properties in PT and SG had a stronger influence on bacterial communities. Soil EC, pH, SOM and SWC were longer arrows. In Fig 4B, two axes of CAP axis explain 70.34% of total variation and soil EC (*P* = 0.004), pH (*P* = 0.022), SOM (*P* = 0.019) and SWC (*P* = 0.017) were closely correlated to the CAP axis. As for SG, two axes of CAP axis explain 58.59% of total variation, and soil EC (*P* = 0.023), pH (*P* = 0.017) and SOM (*P* = 0.019) were closely correlated to the CAP axis (Fig 4C). We explored the influence of pH, EC, SOM and SWC on bacterial community composition through Linear regression analysis (Fig 4). The PCA plots showed that changes in bacterial communities at phylum levels along PCA1 are closely correlated with SOM and SWC, and indicated that the bacterial community structures are influenced by SOM and SWC. In addition, soil bacterial diversity was significantly affected by pH (Fig 5 and Table 3).

**Table 3:** The correlation coefficients between soil properties and the diversity index.

Diversity Index	EC	pH	SOM	SWC
Shannon	-0.125	-0.642**	0.116	-0.360
Ace	0.141	-0.802**	0.258	-0.216
Chao	0.081	-0.788**	0.187	-0.249

EC—electrical conductivity; SOM—soil organic matter; SWC—soil water content.

**P < 0.01.

### Soil potential bacterial functions

**Fig 6:**
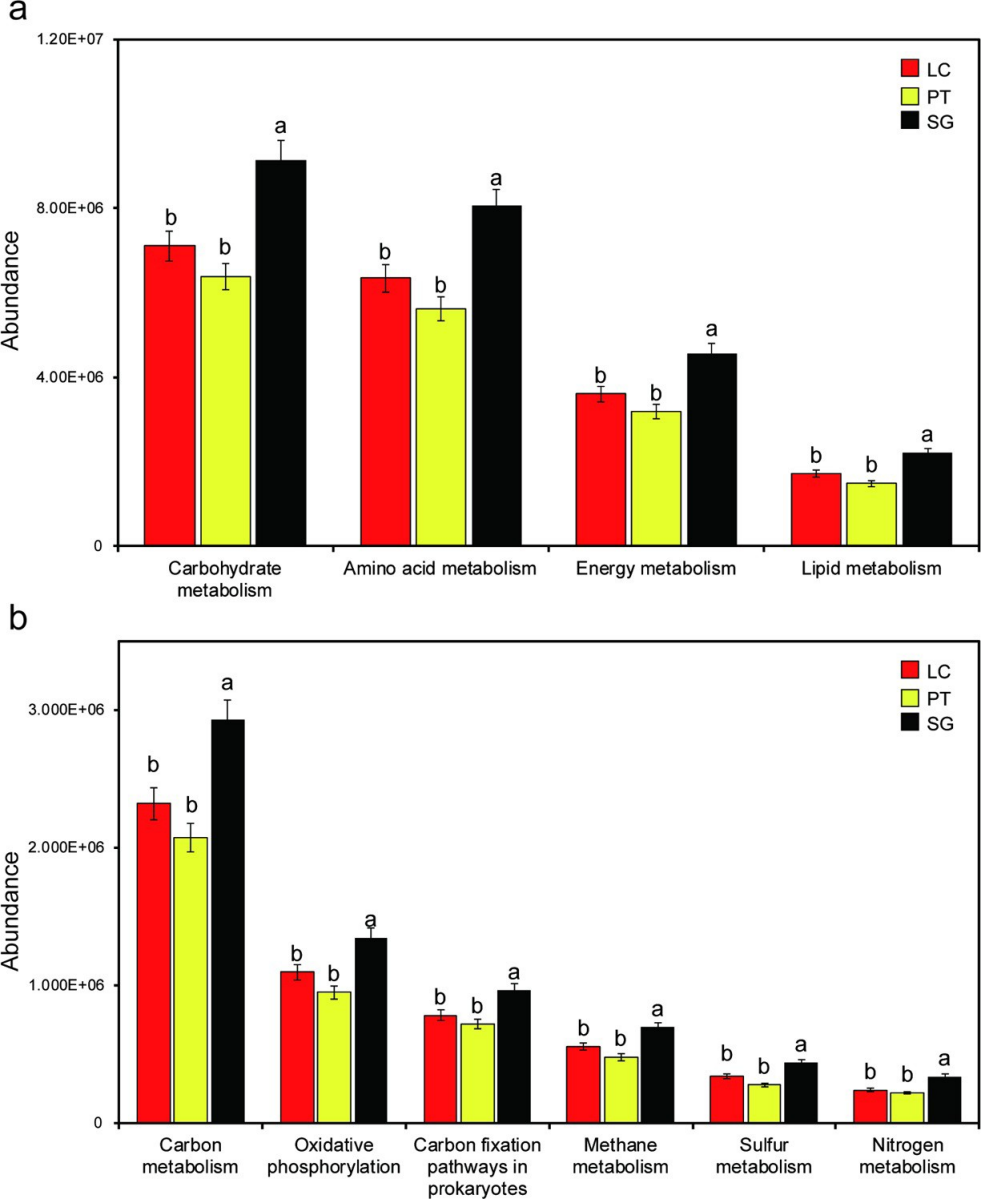
Predicted functions of the soil bacterial community using PICRUSt at KEGG function classification pathway Level2 (a) and pathway Level3 (b). Different lowercase letters into each column are significantly different (Tukey’s test, *P* < 0.05). LC, *Leymus chinensis* (Trin.) Tzvel.; PT, *Puccinellia tenuiflora* (Griseb.) Scribn. et Merr.; SG, *Suaeda glauca* (Bunge) Bunge.

We used PICRUSt to predict the rhizosphere bacterial community function under three halophytes. The classification of KEGG functions at pathway Level1 was performed in S3 Fig. The result showed that the predicted functions mainly the bacterial metabolism at pathway Level1, and the relative abundance of three halophytes was above 78%, and SG was higher than LC and PT. Carbohydrate metabolism, Amino acid metabolism, Energy metabolism and Lipid metabolism were main metabolic functions at pathway Level2 (Fig 6A). Interestingly, the abundance of these metabolism in SG were significantly higher than that in LC and PT (*P* < 0.05, Tukey’s test). At pathway Level3, Carbon metabolism, Oxidative phosphorylation, Carbon fixation pathways in prokaryotes, Methane metabolism, Sulfur metabolism, Nitrogen metabolism were no significant difference between LC and PT (Fig 6B, *P* > 0.05), which was significantly lower than those in SG (*P* < 0.05).

## Discussion

### Soil properties

Plants, rhizosphere bacterial communities and soil properties have a complex micro-ecological relationship, any changes may affect the bacterial structure and ecological processes [32]. Our study found that soil properties, such as pH, EC and SOM were changed significant in soil sample under three halophytes rhizosphere. Soil pH in *Puccinellia tenuiflora* was significantly higher than that of *Leymus chinensis* and *Suaeda glauca*. The changes of soil pH under three halophytes rhizosphere may be due to two reasons. The first is changes in soil pH attributed to the different concentration of CO_3_^2−^ and HCO_3_^−^ in soil [33]. The second is the organic acid secreted by plant roots and metabolites of rhizosphere microorganisms [13, 25]. Higher soluble salt content leads to the higher EC in *Leymus chinensis* than that in *Suaeda glauca* and *Puccinellia tenuiflora*. The reason for the decline EC in *Suaeda glauca* rhizosphere is succulent halophytes can absorb soil salt into their succulent leaves [34]. Soil organic matter was influenced by different halophytes type [35]. We found that SOM content is significant differences from sample under three halophytes rhizosphere, and the highest SOM content was observed in *Leymus chinensis*. This discrepancy could be attributed to different halophytes forms influencing organic matter input by plant debris input and rooting depth [36]. Previous studies have confirmed that halophyte root exudates and organic carbon solubilization have a good correlation with the amounts of bacteria [37]. Decay of organic material is primarily a biological process, mainly controlled by moisture, composition and activities of soil microorganisms [38]. Schnürer et al. (1986) reported that SWC is important parameters regulating microbial activity in soil [39]. We found that *Leymus chinensis* and *Puccinellia tenuiflora* has a higher SWC. This may promote the process of microorganism-mediated transformation of plant residues into SOM. These results provide further support for the earlier studies that halophytes altered soil characteristics through the joint action of rhizosphere exudates and rhizosphere microorganisms [34, 37].

### Soil bacterial community composition and diversity

The rhizosphere microbes play an important role in promoting plant survival under adverse plant communities [5]. *Proteobacteria*, *Actinobacteria*, *Chloroflexi* and *Gemmatimonadete* were the dominant phyla in this study. These phyla have also been recorded in other halophytes rhizosphere [40]. It is reported that most halophiles were observed in the *Proteobacteria* [41]. Among *Proteobacteria* the *Aeromonas*, *Geobacter*, *Halomonas*, *Burkholderia* and *Azospirillum* are involved in plant growth promotion, antibacterial and antifungal activities [42]. Yamamoto et al. (2018) reported that *Proteobacteria* accounting for over 40% in samples from *Glaux maritima* and *Salicornia europaea* [13]. We found that the relative abundance of *Proteobacteria* was exceed 20% in three halophytes rhizosphere samples (Fig 1A). The highest relative abundance of *Proteobacteria* was observed in *Puccinellia tenuiflora*, with an increase of 0.89% and 1.88% compared to *Leymus chinensis* and *Suaeda glauca*, respectively. The important role of *Actinobacteria* secrete antimicrobial pathogens compounds and *Chloroflexi* decompose organic compounds has been performed in previous studies [43]. In contrast with *Puccinellia tenuiflora*, the relative abundance of *Chloroflexi* in *Leymus chinensis* and *Suaeda glauca* decreased by 0.82% and 1.91%, respectively. However, the relative abundance of *Actinobacteria* was no significant difference in three halophytes rhizosphere samples. A possible explanation that *Actinobacteria* widely present in moderate and high salinity environments [44]. *Arthrobacter* and *Kocuria* were belongs to *Actinobacteria*, which contain cell-wall-bound amino acids [45]. This indicated that enriched *Proteobacteria*, *Actinobacteria and Chloroflexi* in the rhizosphere help three halophytes to adapt to the salty conditions. Plant species certainly affect the structure of bacterial communities, and select specific microbial populations [46]. Specific selection leads to differences in the rhizosphere bacterial communities of halophytes, and confirmed in our research (S1 Fig). *Nitrospirae* and *Cyanobacteria* were significantly enriched in the rhizosphere of *Suaeda glauca*. According to reports, *Nitrospirae* and *Cyanobacteria* play an important role in soil C and N cycle and maintain soil fertility [47]. Currently, plant growth-promoting rhizobacteria have been widely used as biological fertilizers to increase the growth and yield of various crops [48]. These results indicate that rhizosphere bacteria have important assistance for halophytes adapt to saline soil, and the rhizosphere of three halophytes has different enrichment of bacteria. Therefore, we can select different halophytes for planting according to the saline environment, and screen the functional rhizosphere microorganism for development and utilization, which is conducive to the improvement of soil quality and ecological environment.

Previous studies suggested that soil microbial communities are directly affected by halophytes [49]. The NMDS analysis confirmed that soil bacterial community structure was significantly different under three halophytes rhizosphere. This may be related to the roots of different halophytes secrete different chemical substances, which affects the enrichment of bacteria and changes the community structure [50]. The decomposition of root exudates and litter will change soil characteristics and promote the establishment of specific rhizosphere bacterial communities [34]. Bacterial community structure was also affected by soil properties, such as soil EC, pH and SOM [51, 52]. Hence, *Leymus chinensis*, *Puccinellia tenuiflora* and *Suaeda glauca* select for specific bacterial taxa and control microbiomes, which led to differences of bacterial community structure [53]. The composition and structure of the soil bacterial community are the main internal factors affecting the bacterial diversity. The soil bacterial diversity of halophytes rhizosphere in high-salt soil largely depends on the soil properties and plant species [43, 63]. Study has shown that the rhizosphere of plants has specific selectivity for bacteria that colonize the rhizosphere, which change the species richness and homogeneity, leading to differences in alpha diversity [54]. In addition, microbial community diversity also affected by different root exudates [55]. The total number of OTUs and bacterial diversity indices (including Ace, Chao, and Shannon) were the highest in *Leymus chinensis*. This may be related to the higher SOM content and lower pH in the *Leymus chinensis* rhizosphere soil, since accumulation of organic matter increases the chances of successful migration of soil bacteria community [24]. These consequences indicate the *Leymus chinensis* is more conducive to the restoration of soil bacterial diversity in saline-alkali conditions compared with *Puccinellia tenuiflora* and *Suaeda glauca*.

### Correlation between bacterial communities and soil properties

Complex environmental factors determined of vegetation types in different regions, and the soil microbes were particularly sensitive to changes in the external environment. Previous studies suggested that soil properties are the important factors leading to the changes in soil bacterial communities [56]. We found that the rhizosphere bacterial community structures of three halophytes are also influenced by SOM and SWC, which is consistent with findings conducted by Gupta and Drenovsky [48, 57]. This suggested that whether in harsh environmental conditions or in farmland, SOM and SWC are very important for the shaping of soil bacterial community.

It is reported that bacteria biogeography differences are largely controlled by edaphic variables, such as soil pH [58]. Linear regression analysis indicated that Ace, Chao, and Shannon indices negatively correlated with soil pH. This is consistent with prior studies that soil bacterial diversity is affected by pH [59]. The rich bacterial diversity in the rhizosphere soil helps to improve the adaptation of halophytes to saline-alkali stress [60]. Therefore, we can evaluate the saline-alkali stress of halophytes through the rhizosphere bacterial diversity. These findings may help us to understand the interaction between different halophyte rhizosphere bacterial communities and their soil environments.

### Soil bacterial community potential functions

Soil bacteria play a vital part in nutrient cycling, maintaining soil fertility, and carbon sequestration through Amino acid metabolism and Carbohydrate metabolism [61]. Chaudhary et al. (2018) reported that the soil microbial functional gene abundance was affected by different halophyte vegetations [62]. The relative abundance of bacterial metabolism was above 78% under three halophytes rhizosphere in this study. Carbohydrate metabolism, Amino acid metabolism, Energy metabolism and Lipid metabolism were main metabolic functions at pathway Level2. This is consistent with the findings of previous studies that that Amino acid metabolism and Carbohydrate metabolism are the main metabolic functions by PICRUSt predictions [25, 63]. These results also suggested that Amino acid metabolism and Carbohydrate metabolism are main bacterial functions in soil, not only in halophyte vegetation rhizosphere but also in other soil types [64–66]. The C, N and P cycle of halophyte rhizosphere bacteria can be evaluated by the abundance of bacterial functional genes [67]. We observed that Carbon metabolism, Sulfur metabolism and Nitrogen metabolism in *Suaeda glauca* were significantly higher than that in *Leymus chinensis* and *Puccinellia tenuiflora*. This discrepancy may be due to their plant root exudates. One of the manifestations of the rhizosphere effect is that rhizosphere bacteria are strongly affected by plant root exudates, and the metabolites of bacteria will be differences [68]. Pii et al. (2016) showed that plants affected the bacterial function through root exudates as C and N sources [69]. Under the dual influence of rhizosphere bacterial metabolites and root exudates, the soil properties of the halophyte rhizosphere will change [70]. Besides, halophytes improve their ability to adapt to saline-alkali conditions by regulating root exudates and affecting the metabolites of rhizosphere bacteria [38]. We found that SOM and SWC in *Leymus chinensis* and *Puccinellia tenuiflora* are significantly higher than *Suaeda glauca*. According to these data, we can infer that the rhizosphere bacterial community of *Suaeda glauca* adjusts its own metabolism to the arid and barren saline-alkali environment.

## Conclusions

This study has shown that soil properties and rhizosphere bacterial community structure showed significant difference in three halophytes. The highest soil EC and SOM content were found in *Leymus chinensis*. At the phylum level, *Leymus chinensis*, *Puccinellia tenuiflora* and *Suaeda glauca* enriched *Proteobacteria*, *Actinobacteria* and *Chloroflexi* in the rhizosphere to adapt the salt stress. The highest relative abundance of *Proteobacteria* was observed in *Puccinellia tenuiflora*, with an increase of 0.89% and 1.88% compared to *Leymus chinensis* and *Suaeda glauca*, respectively. In contrast with *Puccinellia tenuiflora*, the relative abundance of *Chloroflexi* in *Leymus chinensis* and *Suaeda glauca* decreased by 0.82% and 1.91%, respectively. *Suaeda glauca* rhizosphere has stronger enrichment of *Nitrospirae* and *Cyanobacteria*, and *Leymus chinensis* has higher rhizosphere bacterial diversity. Regression analysis revealed that SOM and SWC have an important influence on rhizosphere bacterial community structure, and pH significantly affected the diversity of bacterial communities. The research has also shown that the rhizosphere bacterial metabolism in *Suaeda glauca* were significantly higher than that in *Leymus chinensis* and *Puccinellia tenuiflora*, which including Carbon metabolism, Sulfur metabolism and Nitrogen metabolism. The limitation of this study is that the rhizosphere samples from different regions with three halophytes are geographically distant, environment and climatic factors are different, and these factors largely affect the diversity of rhizosphere bacterial community. On the other hand, PICRUSt only preliminarily predicted the function of the halophyte rhizosphere bacteria. In future studies, metagenomics and metabolomics should be used for further verification to better understand the functions of different halophyte rhizosphere bacterial communities. In spite of its limitations, the study certainly adds to our understanding of the diversity and functional characteristics of rhizosphere bacterial communities in the typical halophytic vegetation of northern China.

## Supporting information

S1 Fig One-way analysis of variance with Tukey’s honestly significant difference test was conducted to determine the differences between bacterial community at the phylum level (a) and class level (b). Different lowercase letters into each column are significantly different (P < 0.05). LC, Leymus chinensis (Trin.) Tzvel.; PT, Puccinellia tenuiflora (Griseb.) Scribn. et Merr.; SG, Suaeda glauca (Bunge) Bunge.
(DOCX)

S2 Fig Venn diagram showing unique and shared OTUs under rhizosphere soil of three halophytes. LC, *Leymus chinensis* (Trin.) Tzvel.; PT, *Puccinellia tenuiflora* (Griseb.) Scribn. et Merr.; SG, *Suaeda glauca* (Bunge) Bunge.
(DOCX)

S3 Fig Pie chart showing the classification of KEGG functions at pathway Level1 under rhizosphere soil of three halophytes. LC, *Leymus chinensis* (Trin.) Tzvel.; PT, *Puccinellia tenuiflora* (Griseb.) Scribn. et Merr.; SG, *Suaeda glauca* (Bunge) Bunge.
(DOCX)

S1 Table Correlation between factors parameters and Db-RDA axes. SOM—soil organic matter; SWC—soil water content. LC, *Leymus chinensis* (Trin.) Tzvel.; PT, *Puccinellia tenuiflora* (Griseb.) Scribn. et Merr.; SG, *Suaeda glauca* (Bunge) Bunge.
(DOCX)
