# Supplementary material for: Rhizosphere bacteria community and functions under typical natural halophyte communities in North China salinized areas
Source: PLoS One. 2021 Nov 11;16(11):e0259515. doi: 10.1371/journal.pone.0259515 (PMC8584676; doi:10.1371/journal.pone.0259515)
Supplement: S1 Fig — One-way analysis of variance with Tukey’s honestly significant difference test was conducted to determine the differences between bacterial community at the phylum level (a) and class level (b). Different lowercase letters into each column are significantly different (P < 0.05). LC, Leymus chinensis (Trin.) Tzvel.; PT, Puccinellia tenuiflora (Griseb.) Scribn. et Merr.; SG, Suaeda glauca (Bunge) Bunge. (DOCX) [file pone.0259515.s001.docx]

**
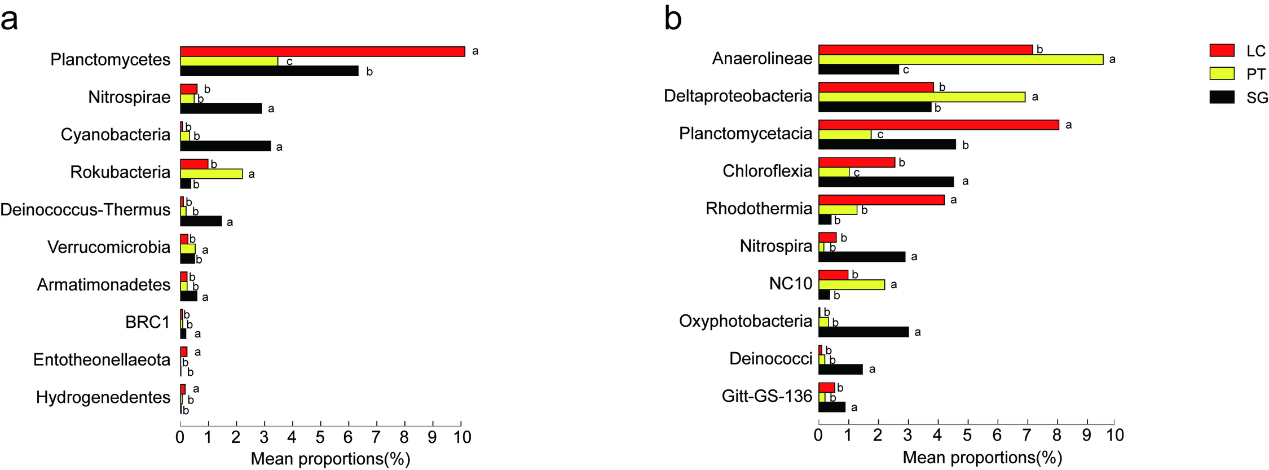
**

**S1 Fig.** **One-way analysis of variance with Tukey’s honestly significant difference test was conducted to determine the differences between bacterial community at** **the phylum level (a) and class level (b)**. Different lowercase letters into each column are significantly different (*P* < 0.05). LC, *Leymus chinensis* (Trin.) Tzvel.*;* PT, *Puccinellia tenuiflora* (Griseb.) Scribn. et Merr.*;* SG, *Suaeda glauca* (Bunge) Bunge.
