# Supplementary material for: Rhizosphere bacteria community and functions under typical natural halophyte communities in North China salinized areas
Source: PLoS One. 2021 Nov 11;16(11):e0259515. doi: 10.1371/journal.pone.0259515 (PMC8584676; doi:10.1371/journal.pone.0259515)
Supplement: S2 Fig — LC, Leymus chinensis (Trin.) Tzvel.; PT, Puccinellia tenuiflora (Griseb.) Scribn. et Merr.; SG, Suaeda glauca (Bunge) Bunge. (DOCX) [file pone.0259515.s002.docx]

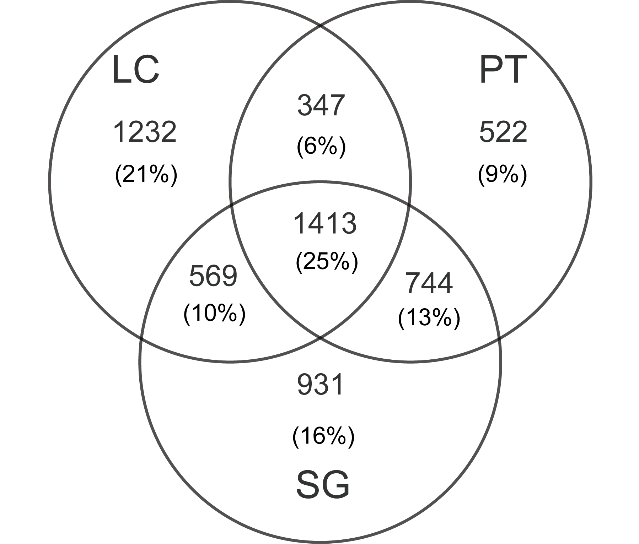


**S2 Fig. Venn diagram showing unique and shared OTUs under** **rhizosphere soil of** **three** **halophytes.** LC, *Leymus chinensis* (Trin.) Tzvel.*;* PT, *Puccinellia tenuiflora* (Griseb.) Scribn. et Merr.*;* SG, *Suaeda glauca* (Bunge) Bunge*.*
