# Supplementary material for: Rhizosphere bacteria community and functions under typical natural halophyte communities in North China salinized areas
Source: PLoS One. 2021 Nov 11;16(11):e0259515. doi: 10.1371/journal.pone.0259515 (PMC8584676; doi:10.1371/journal.pone.0259515)
Supplement: S1 Table — SOM—soil organic matter; SWC—soil water content. LC, Leymus chinensis (Trin.) Tzvel.; PT, Puccinellia tenuiflora (Griseb.) Scribn. et Merr.; SG, Suaeda glauca (Bunge) Bunge. (DOCX) [file pone.0259515.s004.docx]

| **S1 Table**  Correlation between factors parameters and Db-RDA axes. | | | | | | | | | | | | | | |
| --- | --- | --- | --- | --- | --- | --- | --- | --- | --- | --- | --- | --- | --- | --- |
| Factors | LC | | | |  | PT | | | |  | SG | | | |
|  | CAP1 | CAP2 | *R*^2^ | *P* values |  | CAP1 | CAP2 | *R*^2^ | *P* values |  | CAP1 | CAP2 | *R*^2^ | *P* values |
| EC | 0.2834 | -0.959 | 0.1473 | 0.644 |  | 0.8547 | 0.519 | 0.9419 | **0.004** |  | -0.9736 | 0.2284 | 0.7178 | **0.023** |
| pH | 0.419 | 0.908 | 0.4065 | 0.182 |  | 0.7345 | 0.6786 | 0.8512 | **0.022** |  | -0.9681 | 0.2506 | 0.7722 | **0.017** |
| SOM | -0.3735 | 0.9276 | 0.1606 | 0.571 |  | -0.4468 | -0.8946 | 0.8172 | **0.019** |  | -0.9998 | 0.0193 | 0.7405 | **0.017** |
| SWC | -0.057 | 0.9984 | 0.0411 | 0.858 |  | -0.4174 | -0.9087 | 0.8486 | **0.017** |  | -0.1762 | 0.9844 | 0.3826 | 0.206 |

SOM - soil organic matter; SWC - soil water content. LC, *Leymus chinensis* (Trin.) Tzvel.*;* PT, *Puccinellia tenuiflora* (Griseb.) Scribn. et Merr.*;* SG, *Suaeda glauca* (Bunge) Bunge*.*
